# Prevalence and factors associated with hyperglycemia in a rural population of Tanvè and Dékanmey in Benin in 2019

**DOI:** 10.1371/journal.pgph.0000471

**Published:** 2022-05-26

**Authors:** Nicolas Hamondji Amegan, Ariyoh Salmane Amidou, Corine Yessito Houehanou, Helene Robin, Gwladys Nadia Gbaguidi, Corine Agathe Lucresse Fassinou, Kuassi Daniel Amoussou-Guenou, Pierre-Marie Preux, Philippe Lacroix, Stephan Dismand Houinato, on behalf of TAHES group

**Affiliations:** 1 Ecole Doctorale des Sciences de la Santé, Cotonou, Benin; 2 Laboratoire d’Epidémiologie des Maladies Chroniques et Neurologiques, Cotonou, Benin; 3 Faculté des Sciences de la Santé, Cotonou, Bénin; 4 EpiMaCT—Epidemiology of Chronic Diseases in Tropical Zone, Institute of Epidemiology and Tropical Neurology, Omega Health, Univ. Limoges, Limoges, France; 5 Inserm, U1094, EpiMaCT—Epidemiology of Chronic Diseases in Tropical Zone, Limoges, France; 6 IRD, U270, EpiMaCT—Epidemiology of Chronic Diseases in Tropical Zone, Limoges, France; University of Bern: Universitat Bern, SWITZERLAND

## Abstract

**Background:**

Hyperglycemia leads to serious damage to the body, especially the blood vessels and nerves. This study aimed to determine the prevalence and factors associated with hyperglycemia in a rural population of Tanvè and Dékanmey in Benin in 2019.

**Materials and methods:**

This was a cross-sectional, descriptive and analytical study, nested in the Tanvè Health Study (TAHES) cohort. It covered all residents of the villages of Tanvè and Dékanmey, aged 25 years and above, and having given their written consent. Data were collected in the households during the fourth annual monitoring visit in 2019 using the WHO STEPS Wise approach. Hyperglycemia was defined as a fasting capillary blood glucose value ≥ 110 mg/dL. Data were analyzed with R Studio software version 3.5.1.

**Results:**

A total of 1331 subjects were included in the study with a 60% female predominance and a sex ratio (male/female) of 0.7. The median age was 40 years (Q1 = 32 years; Q3 = 53 years) with a range of 25 and 98 years. The prevalence of hyperglycemia was 4.6%. In multivariate analysis, advanced age (AOR = 1.03; 95%CI = 1.02–1.73; p = 0.004), male sex (AOR = 2.93; 95%CI = 1.49–5.84; p = 0.023), monthly income> 105,000 FCFA (AOR = 2.63; 95%CI = 1.24–5.63; p = 0.030), abdominal obesity (AOR = 2.80; 95%CI = 1.29–6.16; p = 0.007, and obesity (AOR = 1.68; 95%CI = 0.75–3.59; p = 0.004) were statistically associated with hyperglycemia.

**Conclusion:**

The prevalence of hyperglycemia is not negligible in rural areas in Benin. Our study found that older age, male gender, high income, abdominal obesity, and obesity are determining factors in its occurrence.

## Background

Hyperglycemia is a precursor stage of diabetes, which corresponds to an increase in fasting glucose from 110 mg/dL [1].

The International Diabetes Federation (IDF) estimated in 2013 the number of adults suffering from diabetes to around 382 million in the world, meaning a global proportion of 8.3%, with around 80% of these people living in Low and Middle Income Countries (LMICs). If nothing is done, by 2035, the number of persons living with diabetes will rise to around 592 million; meaning one in ten adults. Estimates based on these same figures, predict about three new cases every ten seconds, which corresponds to nearly 10 million per year [2].

The World Health Organization (WHO) warned in 2012 that hyperglycemia caused around 2.2 million deaths in the world while diabetes was the direct cause of 1.6 million deaths a few years later in 2015 [1]. In addition, almost half of all deaths due to diabetes have been recorded in population under 60 years of age, and mostly in less developed regions such as Sub-Saharan Africa (SSA), where 75% of the global deaths occurred [2].

In general, hyperglycemia and diabetes result in disabilities due to their manifestations and the expensive cost of treatment. The IDF estimated in 2017 that two-thirds of people ($327 million) living with diabetes are of working age; adding that in the same year, diabetes was the cause of $727 billion in health spending [3]. Hyperglycemia has a serious impact on human health and constitutes an independent cardiovascular risk factor [4]. It causes as many deaths, all causes combined, as smoking or increased cholesterol and blood pressure [5]. Moreover, considered as a potentially modifiable factor, it is associated with an unfavorable progression of stroke in acute phase, even in the absence of known diabetes (OR = 2.99; 95%CI = 1.37–6.50), also contributing to vascular lesions and complications such as coma, blindness, kidney failure, coronary artery disease and stroke [6].

In the past, high prevalence of hyperglycemia was considered to be the prerogative of high-income urban populations; but today, studies show significant prevalence of hyperglycemia in rural areas and according to IFD in 2013, more people with diabetes live in rural areas, meaning 136 million compared with 246 million in urban areas [2]. LMICs are not on the sidelines of the question. In fact, in 2013, the number of people living with diabetes in rural areas was 122 million compared to 181 million in urban areas and by 2035, estimates predict 145 million diabetics in rural areas versus 347 million people in urban areas [2].

The situation is no less worrying in rural areas in Benin as evidenced by the Tanvè Health Study (TAHES) cohort erected as a rural population in South Benin since 2015 where prevalence of hyperglycemia has been estimated to respectively 3.5% and 4.5% in 2015 and 2017 [7, 8]. In light of this statistics, it is quite imperative to act quickly to curb the increase in the burden of hyperglycemia in order to prevent its harmful consequences on the health of rural populations. Our research aimed then, to study the factors associated with hyperglycemia in rural areas in Benin in order to guide the implementation of possible consequent control actions.

## Materials and methods

Our study was a cross-sectional, descriptive and analytical, conducted during the fourth annual follow-up visit of the TAHES cohort (Tanvè Health Study). The study population included persons aged 25 years or older, residing in the villages of Tanvè and Dékanmey, and having given their free written consent after clarification to participate in the TAHES cohort.

Fasting hyperglycemia was considered as the dependent variable and was defined by fasting capillary glycemia ≥110 mg/dL. The glycemia was defined as a diabetic-type in case of fasting capillary glycemia ≥126 mg/dL The independent variables were socio-demographic, economic, cultural, behavioural and biological variables. The definition of risk factors used in our study was those described in the WHO STEPS Survey Manual. Only current and former smokers were considered to be smokers. Consumption of less than five servings (400 grams) of fruits and vegetables (FV) per day was defined as insufficient consumption of fruits and vegetables. The practice of physical activity was categorized in 2 ways: sufficient and insufficient. Subjects were reported to have insufficient physical activity when they had moderate physical activity of at least 30 minutes or intense activity of at least 15 minutes during their activities or recreation for less than 5 days per week. This practice was considered as sufficient when it was done for at least 5 days a week. Alcohol consumption has been defined as a consumption on at least one occasion in the past 12 months. Without any notion of follow-up, systolic blood pressure ≥ 140 mm Hg and/or diastolic blood pressure ≥90 mm Hg was defined as High Blood Pressure (HBP). Hypertension has been defined as either having HBP or being on antihypertensive treatment. The body mass index (BMI) was calculated by dividing the weight in kilograms (Kg) by the square of the height in meters (m). Under-weight was defined as a BMI <18.5kg/m^2^, overweight by a BMI comprised between 25kg/m^2^ and <30kg/m^2^ and obesity as BMI ≥ 30kg/m^2^. Abdominal obesity was defined by a waist circumference of at least 80 cm in women and 94 cm in men according to IFD criteria.

The data were collected from 26th January to 17th February 2019 in the habitations using the WHO STEPS Wise approach. An individual door-to-door interview was done with each respondent (STEP1), with anthropometric measurements (weight, height, waist circumference) and blood pressure that were after the interview, taken according to WHO standards with validated materials (STEP2). Capillary glucose was measured by finger on appointment the next morning after at least 8 hours of fasting using a glucose meter. The data were recorded using a standardized sheet adapted from the WHO STEPS instrument and integrated into the smartphone via the Kobo-collect application. The study was approved by the National Ethics Committee for Health Research and pregnant women were excluded from the study.

The collected data were cleared and analyzed with R Studio 3.5.1 software. The proportions were compared with the Chi-two test; the means with the Student test and the medians with the non-parametric Kruskal-Wallis test. A step-by-step descending binary logistic regression of Wald was performed using a significance threshold p < 20% for initial model and p < 5% for final model. Prior to model execution, the interactions were verified and the suitability of the model was made by the Akaike Information Criterion (AIC).

## Results

A total of 1331 subjects were included in the study. The median age was 40 years (Q1 = 32 years; Q3 = 53 years) with extreme values of 25 and 98 years. There was a female predominance (60%) with a sex ratio of 0.7. In the sample, 63.4% had no educational level, 40.1% had a monthly income of 40,000 FCFA. Poor fruit and vegetable consumption was found in 96.4%, alcohol consumption in 63.9%, tobacco consumption in 5.0% and insufficient physical activity in 62.5%. Concerning the biological risk factors, the average waist circumference was specifically 83.4 ±10.8 cm (extremes: 53 cm and 136 cm) in male subjects and 86.8 ±12.6 cm (extremes: 52 cm and 162 cm) in female subjects. The average BMI was 23.3 ±5.2 Kg/m2.

Abdominal obesity was found in 49.6% of the population. Almost one in ten persons (9.8%) was found to be obese and 19.5% of them were overweight. High blood pressure was found in 29.5% and hypertension was found in 32.1%. In addition, around 2 in 10 persons (21.1%) reported taking a glycemic control less than one year ago while 78.9% of them reported doing so within a year or more. A total of 61 subjects had fasting hyperglycemia, meaning a prevalence of 4.6% (IC95%: 3.6–5.8). The average fasting blood glucose value was 93.5 ±33.8 mg/dL with extreme values of 51.0 mg/dL and 333.0 mg/dL. This value was 85.6 ±36.3 mg/dL in male subjects and 82.2 ±31.9 mg/dL in female subjects (p = 0.073). On the other hand, 2.6% had diabetic-type hyperglycemia and 3.8% had a known history of diabetes. The Behavioral and Biological Risk Factors are described in Tables 1 and 2 below. After binary logistic regression, factors independently associated with hyperglycemia were: advanced age, male sex, abdominal obesity, weight status and monthly income above 105,000 FCFA (Table 3).

**Table 1:** Distribution of surveyed subjects according to the behavioral risk factors in rural population of Tanvè and Dékanmey in Benin in 2019 (N = 1331).

	Frequency (N = 1331)	Percentage (%)
**Fruits and vegetables consumption**		
Sufficient	48	3.6
Insufficient	1283	96.4
**Add salt to meal after cooking**		
Yes	220	16.5
No	1111	83.5
**Alcohol consumption**		
Yes	850	63.9
No	481	36.1
**Tobacco consumption**		
Yes	66	5.0
No	1265	95.0
**Physical activity**		
Sufficient	499	37.5
Insufficient	832	62.5

**Table 2:** Distribution of surveyed subjects according to biological risk factors in rural population of Tanvè and Dékanmey in Benin in 2019 (N = 1331).

	Frequency (N = 1331)	Percentage (%)
**Abdominal obesity**		
Yes	660	49.6
No	671	50.4
**Weight status (BMI)**		
Under-weight	195	14.7
Normal	746	56.0
Over-weight	259	19.5
Obesity	130	9.8
**High Blood pressure**		
Yes	393	29.5
No	938	70.5
**Hypertension**		
Yes	427	32.1
No	904	67.9

**Table 3:** Final multivariate analysis model of factors associated with hyperglycemia in rural population of Tanvè and Dékanmey in Benin in 2019.

	Bivariate		Multivariate	
	COR [95%CI]	*p*	AOR [95%CI]	*P*
Age (years)	1.02 [1.01–1.04]	***0*.*002***	1.03 [1.02–1.73]	***0*.*004***
**Sex**		***0*.*023***		***0*.*023***
Male	1.82 [1.09–3.07]		2.93 [1.49–5.84]	
Female	1		1	
**Abdominal obesity**		*0*.*077*		***0*.*007***
Yes	1.60 [0.94–2.71]		2.80 [1.29–6.16]	
No	1		1	
**Weight status (BMI)**		***0*.*030***		***0*.*004***
Under-weight	0.55 [0.22–1.38]		0.58 [0.18–1.46]	
Normal	1		1	
Over-weight	0.70 [0.36–1.52]		0.48 [0.19–1.04]	
Obesity	1.97 [1.05–3.69]		1.68 [0.75–3.59]	
**Monthly income (FCFA)**		***0*.*047***		***0*.*030***
< 40,000	1		1	
40,000–105,000	1.75 [0.94–3.35]		1.74 [0.90–3.46]	
> 105,000	2.31 [1.16–4.66]		2.63 [1.24–5.63]	

## Discussion

### Prevalence of hyperglycemia and diabetes

We found in our study a prevalence of fasting hyperglycemia of 4.6% (95%CI: 3.6–5.8). This prevalence corroborates those of Amidou et al and Houehanou et al who respectively found within the same cohort in 2017 and 2015, a prevalence of 4.5% and 3.5% [7, 8]. Also, the STEPS survey realized in Burkina Faso in 2013 showed a similar result with a prevalence of hyperglycemia of 4.9% [9]. In contrast to the results found in our study, studies realized through the STEPS surveys in Senegal in 2015 and Congo Brazzaville in 2014 showed respectively a prevalence of 2.1% and 7.1% [10, 11]. Moreover, in Uganda in 2014, Bahendeka et al, according to a national survey, reported a prevalence of fasting hyperglycemia of 1.9% (IC95%: 1.3–2.4) in rural areas, a result that is lower than ours [12].These differences could be first explained by a disparity in dietary and life behaviors of rural populations in these countries. In addition, the differences observed in the inclusion criteria such as age could be part of these disparities as it has been established that the prevalence of hyperglycemia increases significantly with age. In fact, in some studies, the minimum age required for inclusion was 18 while it was 25 in others.

The prevalence of diabetic-type hyperglycemia in our study was 2.6% and that of diabetes was 3.8%. This result is similar to that obtained by Fen L et al in China in a study of 2,967 residents of the Han ethnic group aged 20 to 80 years, selected by multi-stage cluster sampling in Guizhou and which reported a prevalence of 3.77% among rural residents [13]. Bahendeka et al also reported in their study a prevalence of 1% (95%CI: 0.5–1.6) for diabetic hyperglycemia in rural settings [12]. The prevalence of diabetes found in our study was 3.8%; which was lower than that of Mohamed et al in Kenya who reported a prevalence of 1.9% in rural areas in 2015. This slight difference could be explained by the socio-cultural disparities and lifestyle patterns of the two study environments [14].

Our study also revealed that 281 (21.1%) persons reported having taken a glycemic control less than a year ago whereas 1050 (78.9%) of them reported having taken it more than a year ago. These results showed that this rural population is becoming more aware of its health. In fact, the 21.1% who did this check-up, were supposed to have done it in the absence of an annual check-up visit as part of the TAHES Cohort since the last visit before that of our study had been done in 2017. Similar to our findings, BeLue et al in Senegal reported in their study a proportion of 24.8% in type 2 diabetic patients that were regular in taking their glycemic control at the M’Bour Hospital Complex [15]. On the contrary, Mohamed et al in Kenya in 2015, based on STEPS data, reported that out of a total of 4,069 respondents aged 18 to 69, only 43.7% knew their glycemic status [14]. The difference with our results could be explained by the fact that their study was not specific to the rural environment. Moreover, the results of the STEPS survey of Burkina Faso in 2013, showed a prevalence of 5.8% for glycemic control [9]. Another factor that could influence the glycemic control is pregnancy. In fact, pregnant women often benefit in the context of their prenatal consultations, from a series of routine tests that include glycemic control. As a result, when these women are part of the next survey concerning the cohort TAHES as they are no longer pregnant, they will report having carried out a glycemic control, although this control was not a voluntary control, what could over-estimate the prevalence of voluntary glycemic control in the population. Also, the exclusion of pregnant women from the study could underestimate the prevalence of glycemic control as these women that were not pregnant the previous year, were subject to a glycemic control during the previous survey and could have reported a glycemic control if considered in the current study.

### Factors associated with fasting hyperglycemia

In our study, factors associated with fasting hyperglycemia in multivariate analysis were: advanced age, male sex, abdominal obesity, weight status and high monthly income.

The prevalence of hyperglycemia increased significantly with age (p = 0.004). In fact, the risk of being hyperglycemic increased by 1.03 with each increase of one unit of age (95%CI: 1.02–1.73). Bahendeka et al had reached out to the same conclusion in their study where they reported that the prevalence of hyperglycemia increased significantly with age (p = 0.003) [12]. Furthermore, our study revealed that men were at a higher risk of developing hyperglycemia compared to women (p = 0.023), AOR = 2.93 (1.49–5.84). Okumiya et al also found a significant association between hyperglycemia and sex in Tibetans in Asia. In their study, hyperglycemia was 2.17 times more frequent in men than in women (95%CI AOR: 1.57–2.99; p < 0.0001) [16]. The same observation was made by Saleem et al in the United States [17]. Feng et al had come to the same conclusion in their study by comparing the mean fasting blood sugar level where they reported that the mean fasting blood sugar level was higher in men than in women (5.23 mmol/L versus 5.09 mmol/L, p = 0.003) [13]. In contrary, Millogo et al in Burkina Faso found that the proportion of hyperglycemia increased significantly in women compared to men (10.4% vs. 6.9%, p < 0.05) [18]. This difference could be explained by the fact that their study took place in urban area where women are less engaged in activities requiring moderate or intense physical activity, contrary to our study environment, which is an almost agricultural environment where the majority of populations, carry out daily rural work and small trade on foot while walking in the surrounding villages, thus deploying a lot of energy. In contrast, Bahendeka et al in Uganda did not find a significant association between hyperglycemia and sex (p = 0.421) [12].

Our study showed that abdominal obesity was associated with hyperglycemia (p = 0.007; AOR = 2.80 (1.29–6.16)); that is consistent with the finding of Aynalem et al in Ethiopia in 2016 who also found an association between hyperglycemia and abdominal obesity. In their study, people with abdominal obesity were more exposed to hyperglycemia compared to people with a normal waist circumference (AOR = 4.107 (1.108–15.231)) [19]. Similarly, Islam et al had the same association in Bangladesh in a study with a representative sample of 1,843 adults aged at least 18 [20].

Obesity is established as an associated factor of hyperglycemia. In our study we found a significant association between hyperglycemia and obesity in univariate analysis (COR = 1.97(1.05–3.69); p = 0.030)). After adjustment, this significant association remained, though unstable (AOR = 1.68 (0.75–3.59); p = 0.004). Okumiya et al reported in their study that obese people were 1.77 times more exposed to hyperglycemia compared to people with normal BMI (95%CI: 1.33–2.36; p < 0.0001) [16]. Millogo et al also reported in their study that hyperglycemia was two times more frequent in obese or overweight subjects compared to normal or under-weight subjects (13.3% vs 5.9%, p < 0.00001) [18]. Similarly, Binh et al in rural population in Vietnam; out of a sample of 2710 people, had come to the conclusion that obesity is a factor associated with hyperglycemia (OR = 2.41, 95%CI:1.41 4.11) [21].

People with relatively high incomes tend in a given society to adopt eating habits or lifestyle habits in general, similar to a life of ease. The latter can sometimes lead to the occurrence of hyperglycemia which can progress to diabetes. Our study found that the prevalence of hyperglycemia was significantly higher among participants with a monthly income above 105,000 FCFA, compared to those with a monthly income below the Beninese’s minimum wage (< 40,000 FCFA). Furthermore, hyperglycemia was 2.63 times more frequent in people monthly earning at least the minimum wage, compared to those earning below this amount (p = 0.030; 95%CI AOR: 1.24–5.63). Millogo et al had made the same observation by pointing out in their study that subjects of high socio-economic level were more affected by hyperglycemia (13.6%) than others (7.6%; p = 0.0098) [18]. Harshfield et al’s findings in Bangladesh reinforce this evidence by establishing that the risk of hyperglycemia was four times higher among the richest individuals than among the poorest (OR = 6.48, 95% CI: 5.11–8.22 for men versus 4.77, 95% CI: 3.72–6.12 for women) [22]. Fu et al with 4,506 subjects aged between 18 and 64 concluded that high household income was significantly associated with an increased risk of fasting hyperglycemia in rapidly changing rural Chinese communities (AOR = 1.93 (1.26–2.96); p = 0.002) [23]. On the contrary, Drame et al in Benin in 2015 did not find an association between hyperglycemia and socio-economic status (p = 0.62); which could be explained by the fact that their study area was extended to two administrative departments in Benin [24].

The results of this research must challenge us and draw our attention to a phenomenon that begins to emerge gradually even in the rural regions.

### Strengths and weaknesses of the study

We conducted a comprehensive recruitment of subjects who met the inclusion criteria with the benefit of a large enough sample size. The data were collected by investigators accustomed to the STEPS surveys with the STEPS wise tool of the WHO transcribed for needs in the main language of the medium "fon". Measurements of blood glucose, blood pressure and anthropometric parameters were made by trained investigators using WHO recommended methods and with valid and standardized equipments. All this has contributed to the minimisation of measurement errors and potential information biases.

However, blood sugar and blood pressure measurements being taken during a single visit, are likely to be influenced by external factors such as infection, ongoing treatment, stress or lack of rest; which may overestimate our results.

## Conclusion

The prevalence of hyperglycemia was not negligible in the rural populations of Tanvè and Dékanmey in Benin. Advanced age, males, abdominal obesity, obesity and high income were significantly associated with its occurrence. Effective action against this health problem requires concerted political and health action at a much more multi-sectoral level considering the diverse origins of its risk factors.
